# Widespread and evolutionary analysis of a MITE family *Monkey King* in Brassicaceae

**DOI:** 10.1186/s12870-015-0490-9

**Published:** 2015-06-19

**Authors:** Shutao Dai, Jinna Hou, Yan Long, Jing Wang, Cong Li, Qinqin Xiao, Xiaoxue Jiang, Xiaoxiao Zou, Jun Zou, Jinling Meng

**Affiliations:** National Key Lab of Crop Genetic Improvement, Huazhong Agricultural University, Wuhan, Hubei 430070 China; Crop Designing Centre, Henan Academy of Agricultural Sciences, Zhenzhou, Henan 450002 China

**Keywords:** Brassicaceae, *Brassica*, Miniature inverted repeat transposable elements, *Monkey King*, *Tourist*-like MITE, DNA methylation, bna-miR6031

## Abstract

**Background:**

Miniature inverted repeat transposable elements (MITEs) are important components of eukaryotic genomes, with hundreds of families and many copies, which may play important roles in gene regulation and genome evolution. However, few studies have investigated the molecular mechanisms involved. In our previous study, a *Tourist*-like MITE, *Monkey King*, was identified from the promoter region of a flowering time gene, *BnFLC.A10*, in *Brassica napus*. Based on this MITE, the characteristics and potential roles on gene regulation of the MITE family were analyzed in Brassicaceae.

**Results:**

The characteristics of the *Tourist-like* MITE family *Monkey King* in Brassicaceae, including its distribution, copies and insertion sites in the genomes of major Brassicaceae species were analyzed in this study. *Monkey King* was actively amplified in *Brassica* after divergence from *Arabidopsis*, which was indicated by the prompt increase in copy number and by phylogenetic analysis. The genomic variations caused by *Monkey King* insertions, both intra- and inter-species in *Brassica*, were traced by PCR amplification. Genomic sequence analysis showed that most complete *Monkey King* elements are located in gene-rich regions, less than 3kb from genes, in both the *B. rapa* and *A. thaliana* genomes. Sixty-seven *Brassica* expressed sequence tags carrying *Monkey King* fragments were also identified from the NCBI database. Bisulfite sequencing identified specific DNA methylation of cytosine residues in the *Monkey King* sequence. A fragment containing putative TATA-box motifs in the MITE sequence could bind with nuclear protein(s) extracted from leaves of *B. napus* plants. A *Monkey King*-related microRNA, bna-miR6031, was identified in the microRNA database. In transgenic *A. thaliana*, when the *Monkey King* element was inserted upstream of 35S promoter, the promoter activity was weakened.

**Conclusion:**

*Monkey King*, a Brassicaceae *Tourist-like* MITE family, has amplified relatively recently and has induced intra- and inter-species genomic variations in *Brassica. Monkey King* elements are most abundant in the vicinity of genes and may have a substantial effect on genome-wide gene regulation in Brassicaceae. *Monkey King* insertions potentially regulate gene expression and genome evolution through epigenetic modification and new regulatory motif production.

**Electronic supplementary material:**

The online version of this article (doi:10.1186/s12870-015-0490-9) contains supplementary material, which is available to authorized users.

## Background

Miniature inverted repeat transposable elements (MITEs) are a class of non-autonomous DNA transposable elements (classII) [1]. They were first described in the mutated maize allele *wx-B2* [2] and subsequent studies have revealed that MITEs are predominant in almost all plants and animals. They often have terminal inverted repeats (TIRs) and target site duplications (TSDs) at the ends of the elements. Based on TSD sequences, earlier studies showed that MITEs were mainly classified into two super-families: *Tourist*-like MITEs (3-bp, TAA) [2, 3] and *Stowaway*-like MITEs (2-bp, TA) [4]. Studies have shown that MITEs may originate from internal deletion of corresponding autonomous transposable elements; thus, *Tourist* and *Stowaway* MITE super-families are assumed originated from PIF/Harbinger and Tc1/mariner elements, respectively [5–7]. Later studies indicated that some MITEs were derived from other autonomous DNA transospons, such as hAT transposons [8, 9] and Mutator transposons [10]. In addition, due to ambiguous TSD and/or TIR features, Some MITEs were annotated as unknown super-families [11].

There are hundreds of families of MITEs and they are present in high copy numbers, making them important genome constituents. These elements are widely, but not randomly, distributed in the genome, and their distribution density in each chromosome varies [12]. Thousands of MITE copies provide potential resources for genomic structure variation and may fuel genomic evolution. Recent activities of MITEs have produced abundant MITE-derived polymorphisms, which may contribute to considerable phenotypic diversity in rice [13]. *mPing*, a *Tourist*-like MITE, which originated from an internal deletion of a transposase-encoding element *Ping*, is activated by tissue culture and γ-ray irradiation in rice[14–16]. *mPing* insertions presented different profiles (from 50 to 1,000 copies) among four rice strains under selection during domestication [17]. It was suggested that some new alleles induced by *mPing* insertions might benefit the host by creating potentially useful allelic variants and novel, stress-inducible regulatory networks [17, 18].

Although selection pressure tends to eliminate most insertions that reside in gene exons and introns in the early stage of MITE amplification in the genome [17], studies have still found that more ancient MITE subfamilies are preferentially associated with genes [19]. This suggested that MITEs may be associated with the expression of neighboring genes. Much recent research has focused on the function of MITEs in gene regulation. *kiddo*, a MITE located in the rice *ubiquitin2* promoter, has a dual function in gene regulation: its presence not only increases transcription rates but induces epigenetic modifications [20]. Small RNAs regulate the activity of transposable elements via a class of transposable element (TE)-derived 24-nt siRNAs [21]. In Solanaceae, MITEs generate small RNAs that are mostly 24 nt in length and MITE siRNA biogenesis involves *DICER-LIKE 3*, RNA-dependent RNA polymerase 2, and possibly *DICER-LIKE 4* [22].

*Brassica*, a close relative of *Arabidopsis*, is an agriculturally important genus that includes a wide range of diploid and allotetraploid species, including oil crops, vegetables, and forages. *B. napus*, an allotetraploid species (AACC, 2*n* = 2*x* = 38), originated from natural hybridization between the ancestral forms of the diploid species *B. rapa* (AA, 2*n* = 2*x* = 20) and *B. oleracea* (CC, 2*n* = 2*x* = 18) ~ 7500 years ago [23, 24]. The *Brassica* A and C genomes were estimated to have diverged ~ 4.6 million years ago [25]. The same sets of genomes in *B. napus* and its progenitors were defined as subgenomes of each other. The A genomes in *B. rapa* and *B. napus* were assigned the A^r^ and A^n^ subgenomes, respectively; C^o^ and C^n^ represent the C genome in *B. oleracea* and *B. napus*, respectively [26,27]. Sequence-level comparative analysis has revealed that the similarity between the A^r^ and A^n^ subgenomes is 97.5 ± 3.1 %, and is 93.1 ± 4.9 % between the A^r^ and C^n^ subgenomes [28]. It has been suggested that transposable elements contribute to sequence variation in the A and C genomes [23, 28, 29].

A *Stowaway*-like MITE, *BraSto*, first reported in *B. rapa*, was found in the gene space and is still active both in diploid and allotetraploid *Brassica* species [30]. In *B. napus*, a *Tourist*-like MITE, *Monkey King*, was identified in the promoter region of *BnFLC.A10*, a homologue of *Arabidopsis FLOWERING LOCUS C* (*FLC*) [31]. In this study, we found that *Monkey King* elements are not restricted to *Brassica* species, but are specific to the Brassicaceae family. We further investigated its sequence features, distribution, and phylogenetic relationships, and inferred its potential role in the evolution of Brassicaceae genomes. *Monkey King*-related intra- and inter-species polymorphisms were confirmed experimentally. DNA methylation analysis, electrophoretic mobility shift assay (EMSA) analysis, identification of a *Monkey King*-related microRNA (miRNA), and transgenic analysis revealed its effects on gene expression and genome evolution in Brassicaceae.

## Results

### Characteristics of a *Tourist* MITE family, *Monkey King*, in Brassicaceae

**Fig. 1:**
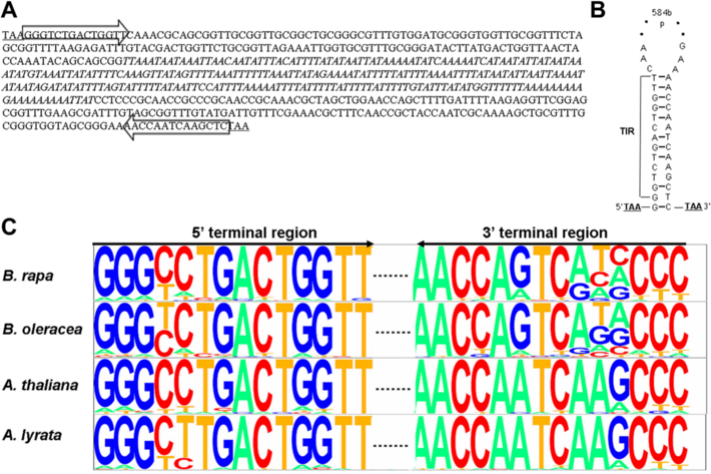
Identification and classification of *Monkey King.* (**a**) Sequence and structural characteristics of the *Monkey King* insertion in the *BnFLC.A10* promoter. The 3-bp TSDs and TIRs are highlighted underlined and framed with arrows at the ends of the sequence, respectively; italics indicate a 270 bp A/T continuous fragment in the core region. (**b**) A Stem-loop structure generated by a pair of 14-bp TIR of the *Monkey King* insertion. Ten of the 14 nucleotides in each of the TIRs are complementary to each other and the other four nucleotides have mismatches. TSDs are underlined. Dots represent the internal sequence in the *Monkey King* insertion. (**c**) Pictogram of TIR sequences obtained from complete *Monkey King* sequences in *B. rapa*, *B. oleracea*, *A. thaliana*, and *A. lyrata*. The height of each letter is proportional to the relative frequency of each nucleotide at that position

The *Monkey King* sequence in the promoter of *BnFLC.A10* included 14 bp TIRs and was flanked with a trinucleotide TAA TSD, which are typical features of *Tourist* MITEs (Fig. 1a). An AT-rich core with a 270-bp A/T continuous fragment was found in the internal region of the sequence. A stem-loop formed in the secondary structure, with the TIRs complementing each other (Fig. 1b). Part of the nucleotide sequence seems to translate into amino acid residues, but no complete protein is encoded (data not shown).

**Fig. 2:**
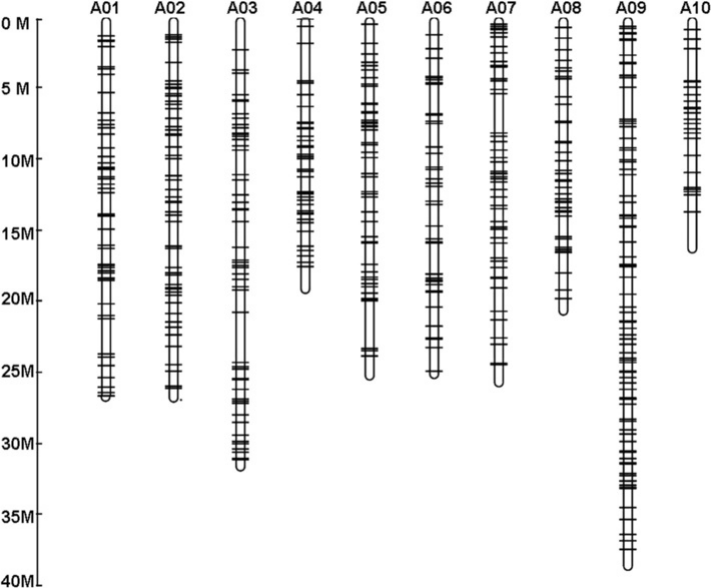
*In silico* mapping of 504 complete *Monkey King* elements in the genome of *B. rapa*. The physical positions details for the *Monkey King* elements are listed in Additional file 1

**Fig. 3:**
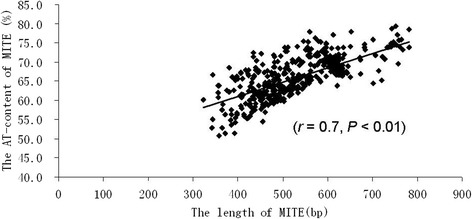
The correlation between the AT-content and the length of complete *Monkey King* sequences in *B. rapa* genome

From the *B. rapa* and *A. lyrata* genome sequences, a total of 1186 and 278 homologous sequences (including complete and partial *Monkey King* sequences), respectively, were screened in the published plant MITE database (P-MITE) [11]. Although no similar sequence was found in the MITE database of *A. thaliana*, 52 *Monkey King* homologous sequences were identified in the *A. thaliana* genome sequence by BLAST analysis (Table 1). *Monkey King* seems to be specific to the Brassicaceae family, because no similar sequences were found in other plant families. *Monkey King* density analysis of the three published genome sequences showed that the *B. rapa* genome, which is the largest genome in size, has the highest density (4.18 MITEs/Mb), while the smallest genome (*A. thaliana*) has the lowest density (0.43 MITEs/Mb). In the same species, no significant differences were found in *Monkey King* density among different chromosomes, except for chromosome 3 from *A. thaliana* and *A. lyrata. In silico* mapping of 504 complete elements on the *B. rapa* chromosomes also showed that they were approximately evenly distributed in their respective chromosomes (Fig. 2). The physical positions of 504 complete *Monkey King* elements from the *B. rapa* genome are listed in Additional file 1. The average length of the complete *Monkey King* sequences varies significantly among the three genome sequences: the shortest was identified in *B. rapa*, followed by *A. lyrata*, while the longest was from *A. thaliana*. The average AT-contents of these sequences vary slightly among the three species (Table 2). However, different *Monkey King* sequences have considerable variation in nucleotide composition in the same genome, especially in the *B. rapa* genome (the AT-content ranged from 50.7 to 79.4 %). Correlation analysis between the AT-content and the length of complete *Monkey King* sequences showed that longer *Monkey King* sequences have relatively higher AT-contents in the *B. rapa* genome ( *r* = 0.7, *P* < 0.01) (Fig. 3).

**Table 1:** Distribution of *Monkey King* elements in *B. rapa, A. lyrata* and *A. thaliana* genome

*B. rapa*				*A. lyrata*				*A. thaliana*			
Chr. no.	Size of Chr. (Mb)	No. of elements	MITE density^a^	Chr. no.	Size of Chr. (Mb)	No. of elements	MITE density	Chr. no.	Size of Chr. (Mb)	No. of elements	MITE density
A01	28.61	134	4.68	Chr.1	33.13	52	1.57	Chr.1	30.43	12	0.39
A02	27.85	131	4.70	Chr.2	19.32	28	1.45	Chr.2	19.70	12	0.61
A03	31.72	139	4.38	Chr.3	24.46	18	0.74	Chr.3	23.46	5	0.21
A04	18.97	101	5.32	Chr.4	23.33	25	1.07	Chr.4	18.59	9	0.48
A05	23.94	110	4.59	Chr.5	21.22	34	1.60	Chr.5	26.98	14	0.52
A06	26.27	113	4.30	Chr.6	25.11	27	1.08				
A07	22.59	119	5.27	Chr.7	24.65	45	1.83				
A08	21.60	94	4.35	Chr.8	22.95	31	1.35				
A09	37.12	184	4.96	Uncertain	12.59	18	1.43				
A10	17.60	62	3.52								
Uncertain	27.58	99	3.59								
Total	283.84	1186	4.18	Total	206.67	278	1.35	Total	119.67	52	0.43

^a^No. of MITEs per Mb

**Table 2:** Nucleotide composition of complete *Monkey King* sequences in *B. rapa, A. lyrata* and *A. thaliana* genomes

Species	No. of complete sequences	The length of complete sequences			The AT-content of complete sequences		
		Min.	Max.	Average	Min.	Max.	Average
*B. rapa*	504	322 bp	791 bp	545 ± 94 bp	50.70 %	79.40 %	67.0 ± 5.0 %
*A. lyrata*	55	452 bp	796 bp	619 ± 46 bp	61.2 %	72.5 %	65.7 ± 2.3 %
*A. thaliana*	38	590 bp	1158 bp	890 ± 150 bp	65.3 %	71.9 %	67.0 ± 1.4 %

*Monkey King* TIR consensus sequences were identified in four Brassicaceae genomes: *B. rapa*, *B. oleracea*, *A. thaliana* and *A. lyrata*. For *B. oleracea*, 70 complete *Monkey King* sequences were identified in the preliminary assembled *B. oleracea* genome sequence using BLAST analysis. The TIR sequences are strongly conserved among these Brassicaceae genomes (Fig. 1c). In general, one specific base occupied the highest proportion for one position. It seems that TIR sequences from the two *Brassica* genomes are more variable than those of the two *Arabidopsis* genomes, especially at the 4^th^ and 5^th^ nucleotides in the 3′ terminal regions. Additionally, there was a distinct difference (A → G transition) at the 9^th^ nucleotide in the 3′ terminal regions between the *Brassica* and *Arabidopsis* genomes.

### Phylogenetic analysis of the *Monkey King* elements in four Brassicaceae genomes

**Fig. 4:**
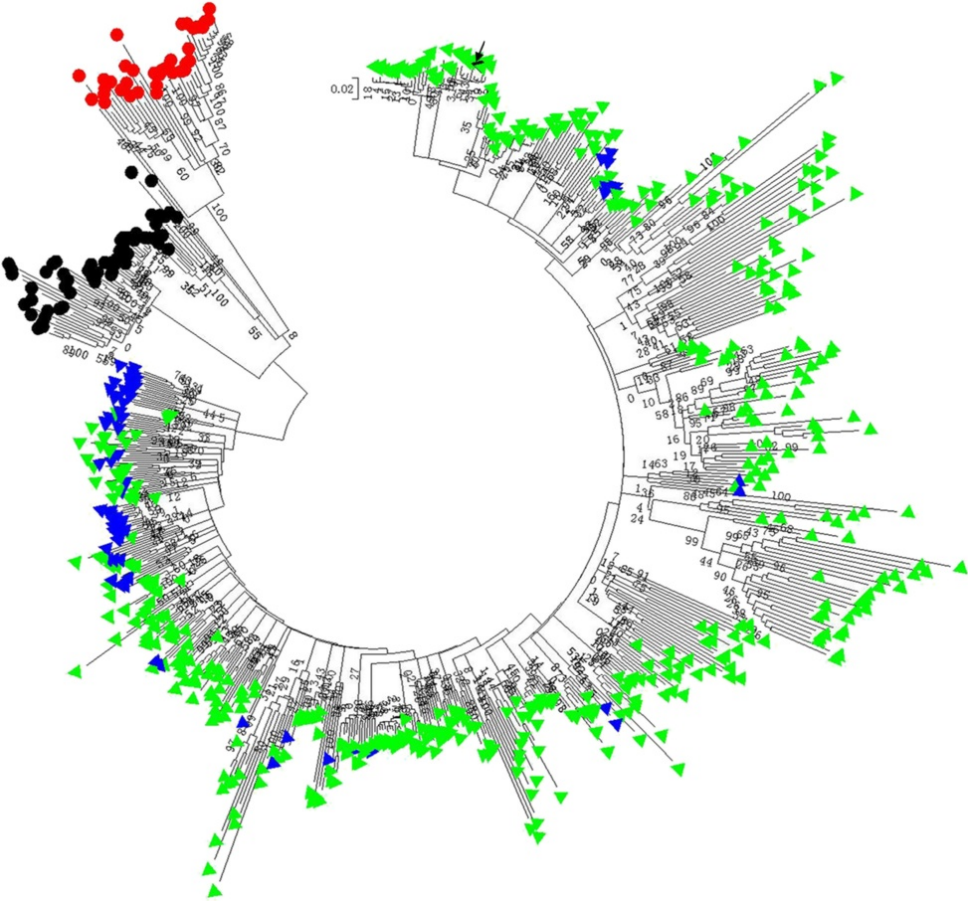
Phylogenetic tree of complete *Monkey King* sequences from Brassicaceae genomes. Red and black circles indicate *A. thaliana* and *A. lyrata Monkey King* sequences, respectively; Green and blue triangles indicate *B. rapa* and *B. oleracea Monkey King* sequences, respectively; the arrow points the *Monkey King* sequence in the *BnFLC.A10* promoter in *B. napus* cultivar Tapidor

All the complete *Monkey King* sequences mined from the four Brassicaceae genomes were used for phylogenetic analysis. In addition, the *Monkey King* sequence in the promoter of *BnFLC.A10* from *B. napus* was included. From the phylogenetic tree (Fig. 4), the *Monkey King* members of *A. thaliana* and *A. lyrata* could be distinguished clearly from the *Brassica* members. By contrast, the *Monkey King* members from the two *Brassica* genomes were interspersed with each other and could not be well separated, which indicated that they have high sequence similarity. However, some members from the same *Brassica* genome formed a small cluster, indicating that they had been rapidly amplified in their respective genomes after A- and C- genomic species differentiation. The *Monkey King* member in the *BnFLC.A10* promoter clustered into an A genome specific group, which indicated that the insertion may be from A genome in *B. napus*. In addition, different small clusters contained *Monkey King* members from *B. rapa* and *B. oleracea*, which indicated that they might have diverged before the differentiation of the A and C genomes.

### The preferred insertion sites of *Monkey King* elements

The insertion sites of the 504 and 38 complete *Monkey King* elements were inspected in the *B. rapa* and *A. thaliana* genomes using the annotated genome databases, respectively. In the *B. rapa* genome, 74.4 % of the elements were inserted in gene-rich regions, less than 3kb from genes. Among them, nearly half of the members were within less than 1kb from a gene, and a few members (24, 4.8 %) were located within introns of genes (Table 3). In the *A. thaliana* genome, notably, 92.1 % of the elements were located in the gene-rich regions, while only three members (7.9 %) were more than 3kb from a gene. Most of the members (26/38) were within less than 1kb from a gene, and two (5.1 %) were within introns (Table 3). We also calculated the distance between the *Monkey King* elements and untranslated regions (UTR) of genes in *A. thaliana* (Additional file 2). 47.3 % of the members (18/38) were within less than 0.5kb from a UTR. Moreover, two members fell within UTR regions. The details of the insertion sites of these complete *Monkey King* elements from the two species are listed in Additional files 1 and 2. Although the *Monkey King* copy ratios in different genomic regions showed some differences between *B. rapa* and *A. thaliana*, a similar trend was observed in the genomic locations between the two species: the closer to a gene, the higher the ratio of *Monkey King* insertions. To further investigate the relationship between *Monkey King* and genes, we examined potential transcriptional activity of *Monkey King* by searching the *Brassica* expressed sequence tag (EST) database at NCBI. Sixty-seven ESTs carrying *Monkey King* fragments were mined from *B. rapa*, *B. oleracea* and *B. napus*. Thirty ESTs matched with annotated *B. rapa* and (or) *A. thaliana* genes (Additional file 3). According to the corresponding gene structure, the *Monkey King* fragments from these ESTs were mainly located in 3′UTR and intron regions. Although more *Monkey King* elements were inserted in the 5′ flanking sequences relative to the 3′ flanking sequences of genes, only one *Monkey King* fragment from a EST was found in a 5′UTR of a gene.

**Table 3:** Summary of the insertion positions of complete *Monkey King* elements in the genomes of *B. rapa* and *A. thaliana*

Insertion position	*B. rapa*		*A. thaliana*	
	No. of elements	Percentage of elements	No. of elements	Percentage of elements
Gene	24	4.8	2	5.3
5′-flank(<1 kb)	110	21.8	18	47.4
5′-flank(1kb to <2 kb)	66	13.1	2	5.3
5′-flank(2kb to <3 kb)	33	6.5	1	2.6
3′-flank(<1 kb)	70	13.9	8	21.1
3′-flank(1kb to <2 kb)	52	10.3	4	10.5
3′-flank(2kb to <3 kb)	20	4.0	0	0.0
intergenic Region (>3 kb)	129	25.6	3	7.9
Total	504	100.0	38	100.0

### Intra- and inter-species polymorphisms caused by *Monkey King* insertions in *Brassica* species

**Fig. 5:**
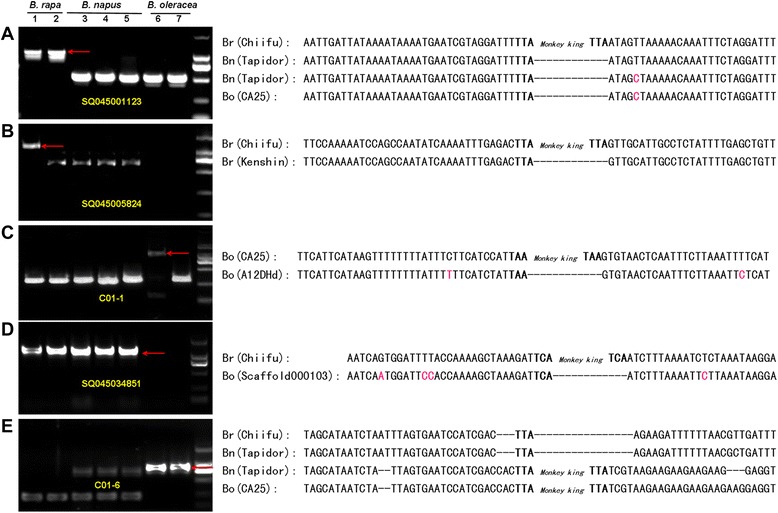
*Monkey King* insertion polymorphism analysis in *B. rapa*, *B. oleracea*, and *B. napus.* Five *Monkey King* members (MITE nos.: SQ045001123, SQ045005824, C01-1, SQ045034851, and C01-6) were used for identification of MITE insertion/deletion in different DNA samples. The Arabic numerals, 1 to 7, represent Chiifu (Br), Kenshin (Br), Tapidor (Bn), Ningyou7 (Bn), Westar (Bn), CA25 (Bo), and A12HDd (Bo), respectively. Red arrows indicate the bands containing MITE insertions and the smaller fragments indicate MITE deletions in the corresponding regions. Sequence comparison information for each *Monkey King* insertion was listed at the right side of the corresponding picture. TSD sequences are shown in bold. Red bases represent base mismatches

To confirm if the *Monkey King* insertions were actually species-specific or cause intra- and inter-species polymorphisms in *Brassica* species, PCR amplification was carried out using primers designed against the *Monkey King* flanking sequences. Sequence comparisons further corroborated the PCR results (Fig. 5). Two *Monkey King* members, SQ045001123 and SQ045005824, were only detected in *B. rapa* and not in *B. napus* or *B. oleracea* (Fig. 5a and b); the *Monkey King* member C01-1 was only observed in *B. oleracea* and not *B. napus* or *B. rapa* (Fig. 5c). Those insertions are probably species-specific and were resulted from independent activation after speciation. The *Monkey King* member SQ045004581 was detected in both *B. rapa* and *B. napus*, but not in *B. oleracea* (Fig. 5d), while the *Monkey King* member C01-6 was detected in both *B. oleracea* and *B. napus*, but not in *B. rapa* (Fig. 5e). We deduced that the two members are A/C genome-specific and were inserted into the *Brassica* genome after *B. rapa* and *B. oleracea* separated from the common ancestor and before *B. napus* speciation. In addition, inter-species polymorphisms caused by *Monkey King* insertions were also observed, e.g. the member SQ045005824 from *B. rapa* and the member C01-6 from *B. oleracea* (Fig. 5b and c).

### *Monkey King* DNA sequence was targeted for methylation and bound by nuclear proteins

**Fig. 6:**
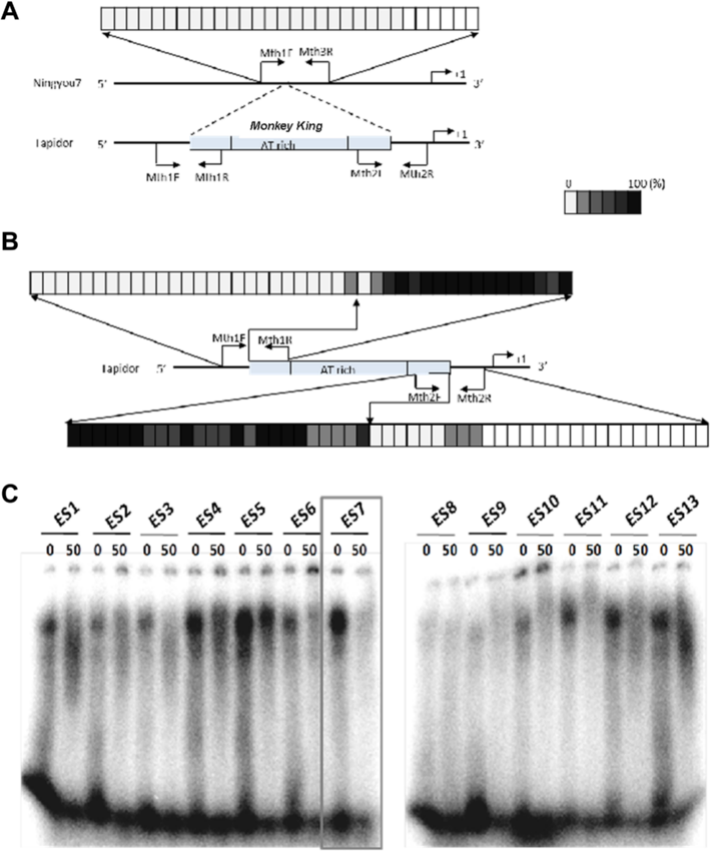
DNA methylation and EMSA analysis of *B. napus Monkey King*. (**a**) and (**b**) DNA methylation detection results in the *Monkey King* sequence and its flanking sequences in the *BnFLC.A10* promoter in Tapidor and Ningyou7. Each cell represents a cytosine. Blank cells denote no methylation. The higher the saturation is, the higher the DNA methylation level. (**c**) EMSA results in Tapidor. Thirteen probes (ES1-ES13) were derived from the *Monkey King* sequence. The binding ability of the probes to nuclear proteins from Tapidor leaves was analyzed by gel shift assays. Plus 50-fold (50) and without plus unlabeled probes (0) were used for the binding assays

The *Monkey King* element identified in the promoter of *BnFLC.A10* in our previous study [31], was used to check the potential ability of *Monkey King* to regulate gene expression via DNA methylation and was subjected to electrophoretic mobility shift assay (EMSA) analysis to check for interacting proteins. The methylation level of cytosine residues inside and flanking the *Monkey King* sequences was investigated using bisulfite sequencing. In *B. napus* cultivar Tapidor, cytosine methylation occurred in the *Monkey King* sequence, while no apparent cytosine methylation was observed in the flanking sequences; in cultivar Ningyou7, no DNA methylation occurred in the corresponding flanking regions (Fig. 6a). This means that DNA methylation was confined strictly to the *Monkey King* sequence.

The EMSA results clearly revealed that nuclear protein(s) extracted from Tapidor leaves specifically bound to a fragment (ES7) from the middle of the *Monkey King* sequence, almost entirely composed of A/T bases (Fig. 6b and Additional file 4). Some *Monkey King* fragments (ES6, ES11, and ES12) were also recognized by nuclear proteins from Tapidor leaves; however, the binding was much weaker than to the ES7 fragment. Some fragments (e.g. ES4 and ES5) were non-specifically bound by nuclear proteins, because retarded bands were observed in both the noncompetitive (without plus unlabeled probes) and competitive (plus 50-fold unlabeled probes) binding assays. This result indicated that the *Monkey King* insertion produced new binding motifs for some nuclear proteins (probably transcriptional factors), which may regulate *BnFLC.A10* expression in winter varieties of *B. napus*.

### Detection of a *Monkey King*-related miRNA

**Fig. 7:**
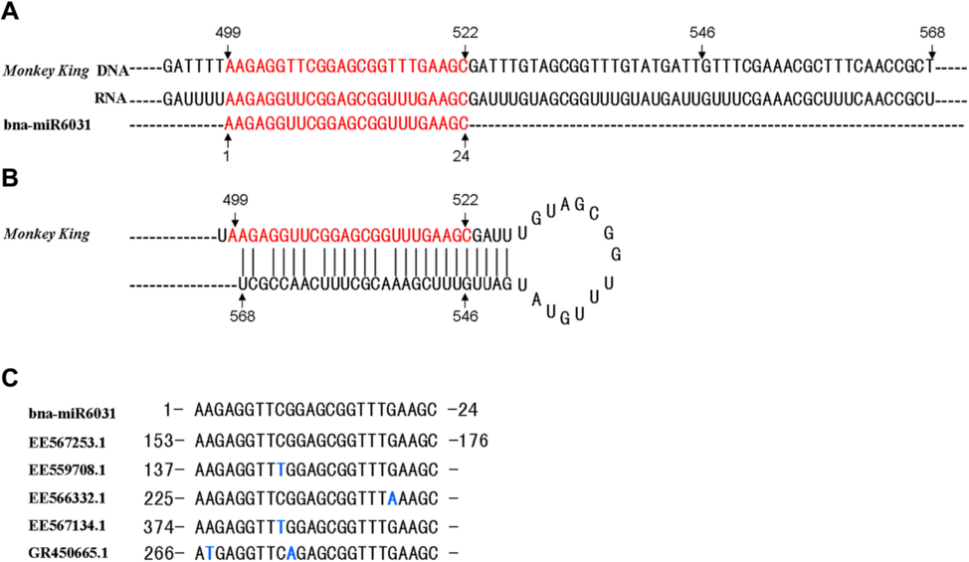
bna-miR6031 generated by *Monkey King* and its potential targets. The numbers and (or) arrows indicate the location of the bases in their respective sequences. (**a**) The alignment of *Monkey King* and bna-miR6031 (miRBase accession no. MIMAT0023651). (**b**) The internal sequence of *Monkey King* can form a bna-miR6031 stem-loop by itself. (**c**) Five ESTs (GenBank accession nos.: EE567253.1, EE559708.1, EE566332.1, EE567134.1, and GR450665.1) from *B. napus* matched perfectly to bna-miR6031. Blue bases represent base mismatches

The *B. napus Monkey King* sequence was used to scan the microRNA database (miRBase) [32] and a miRNA known as bna-miR6031 was found to perfectly match to the internal region (499-522 bases) of the *Monkey King* sequence (Fig. 7a). The miRNA is 24 bp long and was firstly discovered as a new class in *B. napus* by Zhao et al. [33]. Further sequence analysis showed that the internal *Monkey King* sequence (499-586 bases) could form a stem-loop structure by itself (Fig. 7b), which suggested that bna-miR6031 is generated from *Monkey King*. In addition, BLAST analysis revealed that bna-miR6031 aligned well to several *B. napus* ESTs (Fig. 7c). This miRNA was used to search for similar miRNAs in the miRBase; however, none were found in other species, which suggested that bna-miR6031 is a *Brassica* species-specific miRNA.

### The *Monkey King* element decreases the activity of 35S promoter in *A. thaliana*

**Fig. 8:**
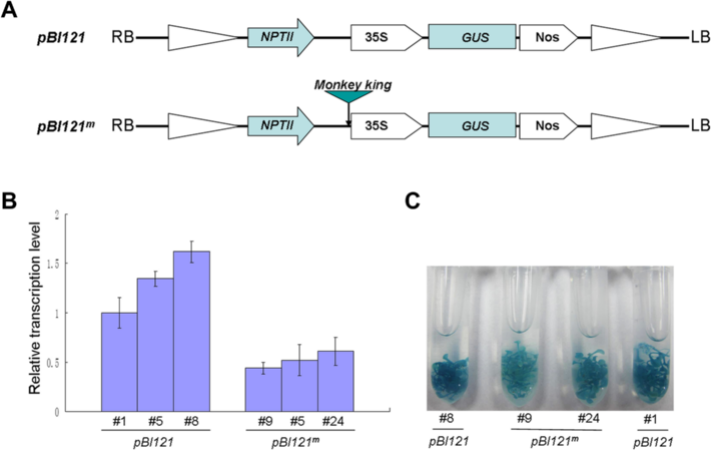
The effect of the *Monkey King* element on the activity of the 35S promoter in transgenic *A. thaliana*. (**a**) Schematic diagram of the pBI121 and pBI121^m^ constructs. The *Monkey King* sequence was inserted upstream of the 35S promoter in the pBI121^m^ construct. (**b**) The relative transcriptional levels of the GUS gene in ten-day-old transgenic *A. thaliana* seedlings carrying the pBI121 and pBI121^m^ constructs, respectively. Error bars represent the standard deviation (n = 3). (**c**) Chemical staining for the GUS activity in ten-day-old transgenic *A. thaliana* seedlings

To further investigate the effect of the *Monkey King* element on gene expression, we studied the influence of the *Monkey King* element on the activity of the 35S promoter that drives a GUS reporter gene. Two expression vectors, pBI121 (without *Monkey King*) and pBI121^m^ (with *Monkey King*) (Fig. 8a) were used to produce transgenic *A. thaliana* via *Agrobacterium*-mediated floral dip transformation. Transcription of the GUS gene was assayed in transgene homozygous T_3_*A. thaliana* seedlings using quantitative real-time reverse transcription PCR (qRT-PCR). Seedlings hosting the pBI121^m^ construct had lower levels of the GUS transcript compared with the pBI121 seedlings (Fig. 8b). Chemical staining also showed that the pBI121^m^ seedlings displayed weaker GUS activities than the pBI121 seedlings (Fig. 8c). These results demonstrated that the *Monkey King* element decreased the activity of the 35S promoter when inserted upstream of the promoter.

## Discussion

In this study, we conducted molecular and genomic characterization of a Brassicaceae *Tourist*-like MITE, termed *Monkey King. Monkey King* possesses all of the typical features of *Tourist* MITEs and has the consensus TIR GGGC(orT)CTGACTGGTT. Interestingly, a similar TIR, GGGGNTGTTTGGTT, is present in *kiddo* and *Tourist-D* in rice and *Hbr* in maize [3, 34, 35]. However, no detectable similarity was observed with the internal sequences of these MITEs. The presence of this TIR in monocot and eudicot MITEs suggested that these MITEs may have evolved from a common ancestor, or that the sequences bearing this TIR easily create new MITE families with dissimilar internal sequences. Transposition of these MITEs may be mediated by the same transposase, because transposases recognize TIRs to ensure MITE mobility across the genome [36]. TIRs of the same family have been used to identify members of a MITE family [19, 37]. TIRs from one species could also be used to find novel MITE families in other species, because different MITE families from different species sometimes contain similar TIRs, such as *Monkey King* from the Brassicaceae family and *kiddo*, *Tourist-D*, and *Hb*r from the grass family.

Comparative analysis of *Brassica* species with *A. thaliana* revealed that, besides genome triplication and allopolyploidization, TEs contribute to the increased genome size of the *Brassica* species [38, 39]. In this study, MITE density analysis showed that *Monkey King* density in the *B. rapa* genome was three and 10 times more than that in the *A. lyrata* and *A. thaliana* genomes, respectively, demonstrating that the elements were actively amplified in the *Brassica* after divergence from *Arabidopsis*. On average, the *B. rapa* genome has the shortest complete *Monkey King* sequence, followed by the *A. lyrata* genome, while the *A. thaliana* genome has the longest sequence. Given the MITE density differences among these Brassicaceae genomes, we inferred that shorter *Monkey King* sequences may be more easily amplified than longer *Monkey King* sequences.

The high conservation of complete *Monkey King* sequences also suggested that they have amplified relatively recently in the Brassicaceae family. The high conservation of TIRs was also considered a sign of recent proliferation in the maize *Tourist* MITE *ZmV1* [7] and the *B. rapa Stowaway* MITE *BraSto* [30]. Some *Monkey King* members have exactly the same TIR sequence, which supports the recent activity and ongoing mobilization of *Monkey King*. This hypothesis is also supported by the phylogenetic relationships among complete *Monkey King* sequences, because some species-specific clusters were found in the phylogenetic tree. In addition, the PCR amplification results from different *Brassica* DNA samples also suggested that some *Monkey King* insertions have arisen from independent activation in each species after speciation. In fact, our previous study suggested that the *Monkey King* insertion in the *BnFLC.A10* promoter occurred in winter rapeseed after *B. napus* speciation [31]. These results indicated that some species-specific *Monkey King* members inserted into their respective genomes independently after allopolyploidization ~7500 years ago.

*Monkey King* is a high copy number *Tourist* MITE found in *B. rapa*, which parallels *BRAMI-1,* a recently identified high copy *Stowaway* MITE [40]. By contrast, In *A. thaliana*, we identified only 52 *Monkey King* members. The physical association between MITEs and genes showed that, in the *B. rapa* and *A. thaliana* genomes, most complete *Monkey King* elements were located in gene-rich regions, less than 3 kb from genes. Many members were within less than 1 kb from a gene, while a few members were within introns. Although the MITE density and copy number showed obvious differences between *B. rapa* and *A. thaliana* genomes, a similar distribution trend was observed in the two genomes: the *Monkey King* sequences were most abundant in the vicinity of genes. The insertion preference of this Brassicaceae *Tourist* MITE is similar to that of the two *Brassica Stowaway* MITEs, *BroSto* and *BRAMI-1* [30, 40]. MITEs inserted in gene regulatory regions can modify gene transcriptional activity and change gene expression levels [13, 41]. In *A. thaliana*, nearly half of the members were within less than 0.5kb from a UTR and two members fell within UTR regions. Additionally, 30 of 67 *Brassica* ESTs carrying *Monkey King* fragments matched the annotated *B. rapa* and (or) *A. thaliana* genes. Thus, *Monkey King* insertions could play a role in gene regulation and evolution in Brassicaceae.

Although many MITEs have been found in plant genomes and are associated with genes, few studies have examined the effects of MITE insertions on neighboring gene expression [18, 20, 22]. In Solanaceae, some MITEs generate small RNAs, thus playing a direct role in gene regulation through the small RNA silencing pathway [22]. In rice, sequences within *mPing* were considered as enhancers that render adjacent genes stress inducible [18]. Promoter activity analysis revealed that the MITE *kiddo* was responsible for up to 20 % of neighboring gene expression in both transient and stably transformed rice calli [20]. Moreover, when *kiddo* DNA methylation was blocked with 5-azaC, *ubiquitin2* transcript accumulation increased threefold [20]. This indicated *kiddo* has a dual function in regulating gene expression. In our previous study, the *Monkey King* insertion upstream of *BnFLC.A10* was positively associated with induced *BnFLC.A10* expression during vernalization [31]. In this study, *Monkey King* DNA methylation in the *BnFLC.A10* promoter was observed. This suggested that epigenetic modification may regulate *BnFLC.A10* expression by changing the *Monkey King* DNA methylation status. *Monkey King* contains important motifs, such as TATA-box and CAAT-box [31]. Certain transcription factors probably bind to the motifs to change neighboring gene expression profiles. Indeed, a fragment (ES7) containing putative TATA-box motifs within *Monkey King* bound nuclear protein(s) extracted from Tapidor leaves. These results suggest that *Monkey King* insertions can produce new regulatory sites, which probably recruit new transcription factors. New alleles caused by MITE insertions may benefit the host by producing useful allelic variants and novel regulatory mechanisms. In fact, *BnFLC.A10* allelic diversity caused by the *Monkey King* insertion is one of the major causes of differentiation of winter and spring *B. napus* genotypes [31]. In addition, identification of the *Monkey King-*related miRNA, bna-miR6031, and its potential targets, indicated that the *Monkey King* silencing pathway may be involved in expression regulation of genes bearing *Monkey King* insertions. In *Arabidopsis*, a transposable element was inserted in an intron of *FLC*, resulting in low *FLC* RNA levels. The intronic TE renders *FLC* subject to repressive chromatin modifications mediated by TE-derived siRNAs [42]. In wheat, a MITE (MITE_VRN) inserted in the promoter of *VRN-A1a* and influenced the flowering [43, 44]. Recent report showed that the MITE_VRN also possessed sequences of a miRNA (TamiR1123) [45]. Whether or not bna-miR6031 regulates *BnFLC.A10* expression through chromatin modifications of the *Monkey King* sequence in the *BnFLC.A10* promoter remains to be investigated. bna-miR6301 is a new class of miRNA found in *B. napus* [33] and may be specific to *Brassica* species because it may be generated by a certain *Monkey King* sequence. Moreover, no similar miRNAs from other species are present in miRbase. To date, many MITEs have only been identified in a given species, which suggests that MITEs can generate species-specific small RNAs. The *Monkey King* insertion was positively associated with *BnFLC.A10* expression [31], however, our transgenic *A. thaliana* experiment revealed that the *Monkey King* element inhibited promoter activity when inserted upstream region of a promoter. These results indicate that a new *Monkey King* insertion may enhance or repress the expression of a gene with which it is associated, by causing DNA epigenetic modification, nuclear protein binding sites and(or) possible miRNA-mediated regulation. Given the wide distribution of *Monkey King* elements in some Brassicaceae genomes, this *Tourist* MITE family may contribute considerable phenotypic diversity to Brassicaceae plants.

## Conclusions

In this study, we characterized a Brassicaceae *Tourist-like* MITE family *Monkey King*. Comparative analysis of *Brassica* species with *Arabidopsis* species revealed its putative role in the evolution of Brassicaceae genomes. Phylogenetic analysis and investigation of intra- and inter-species polymorphisms supported recent proliferation of *Monkey King* in *Brassica* species. *Monkey King* elements are closely associated with genes and influence gene regulation and evolution. DNA methylation detection, EMSA analysis, identification of a *Monkey King*-related miRNA, and transgenic experiments suggested that *Monkey King* insertions could regulate gene expression and genome evolution in the Brassicaceae family in a variety of ways, such as epigenetic modification and new regulatory motif production.

## Methods

### Mining and characterization of *Monkey King* sequences in Brassicaceae genomes

The *Monkey King* sequence inserted in the *BnFLC.A10* promoter was first used as a query to search for similar sequences in the NCBI database and was employed to find its coding capacity. When similar sequences were only found in Brassicaceae genomes, the BLAST program was used to screen the homologous sequences in the published plant MITE databases (P-MITE) [11]. All *Monkey King* sequences in *B. rapa* and *A. lyrata* genomes were downloaded from the P-MITE database for further analysis. *Monkey King* homologous sequences were also identified in *A. thaliana* genome in the NCBI database by BLAST screening (an expected value <1e^−10^) and manual inspection. Additionally, complete *Monkey King* sequences (with TSDs at the two ends of one *Monkey King* sequence) were identified in the preliminary assembled *B. oleracea* genome sequence (http://brassicadb.org/). Nucleotide composition of the complete *Monkey King* sequences was computed in MEGA 5 [46]. Conservation of the TIRs of the complete *Monkey King* sequences among four Brassicaceae plant genomes, *B. rapa*, *B. oleracea*, *A. thaliana* and *A. lyrata*, was visualized using the program Pictograms (http://genes.mit.edu/pictogram.html). Then, the *Monkey King* sequence was used against the microRNA database (miRBase) (http://www.mirbase.org/search.shtml) [32] to find miRNAs.

### Phylogenetic analysis of *Monkey King*

All of the complete *Monkey King* sequences were used for multiple sequence alignment using ClustalW in MEGA 5 [46]. Phylogenetic trees were constructed with the neighbor-joining method in MEGA 5 [46] following manual refinement. Bootstraps with 1000 replicates were performed to assess node support using the p-distance model.

### Characterization of *Monkey King* insertion sites

The insertion sites of 504 and 38 complete *Monkey King* elements and their flanking regions were annotated using the *B. rapa* genome database (http://brassicadb.org/) and the *A. thaliana* genome database (http://www.arabidopsis.org/), respectively. The distances between *Monkey King* elements and their respective nearest genes were calculated for analyzing the relationship between the elements and genes. Additionally, Potential transcription activity of *Monkey King* was examined by searching *Brassica* EST database at NCBI. The *Brassica* ESTs carrying *Monkey King* fragments were used for localizing the *Monkey King* fragments in their respective genes, by comparison with the corresponding annotated *B. rapa* genes (http://brassicadb.org/brad/blastPage.php) and *A. thaliana* genes (http://www.arabidopsis.org/wublast/index2.jsp).

### Survey of intra- and inter-species genomic variations caused by *Monkey King* insertions in *B. rapa*, *B. oleracea*, and *B. napus*

Intra- and inter-species genomic variations caused by *Monkey King* insertions were checked by PCR amplification. The flanking sequences of 5 *Monkey King* members were extracted for primer design (Additional file 5). PCR amplification was performed with DNA samples from two *B. rapa* (Chiifu and Kenshin), three *B. napus* (Tapidor, Ningyou7 and Westar) and two *B. oleracea* (CA25 and A12DHd) accessions. Sequencing and sequence comparisons were performed for further identification of the *Monkey King* insertions.

### DNA methylation detection

Previous studies have identified the *Monkey King* member inserted in the *BnA10.FLC* promoter in winter-type *B. napus* cultivar Tapidor, compared with that in a semi-winter-type *B. napus* cultivar Ningyou7 [31]. Therefore, the two cultivars were used to check the potential ability of *Monkey King* to regulate gene expression.

Genomic DNA was extracted from young Tapidor and Ningyou7 leaves, and 2 μg DNA were subjected to bisulfite treatment using the EpiTect Bisulfite Kit (Qiagen) following the manufacturer’s instructions. The *Monkey King* member in the *BnFLC.A10* promoter was selected to detect the DNA methylation level of its inside and flanking sequences. The primers (Additional file 5) were designed using the software Kismeth (http://katahdin.mssm.edu/kismeth) [47]. Ten clones were sequenced for each amplified product for DNA methylation analysis. The positive control was set to assure that the efficiency of the bisulfite treatment and that all of the non-methylated cytosines were converted to uracil [48].

### Electrophoretic mobility shift assay (EMSA) analysis

Leaves (6 g) were harvested from Tapidor seedlings. Protein extraction was performed as described by Mazabal et al. [49] with some modification. After grinding the leaves in liquid nitrogen, the powder was transferred into 40 ml of homogenizing buffer (10 mM Hepes, pH 7.8, 10 mM KCl, 10 mM MgCl_2_, 5 mM EDTA, 1 mM DTT, 200 mM PMSF, 250 mM sucrose, and 0.5 % Triton X-100) and stirred for over 20 min. The mixture was filtered with a Miracloth and the supernatant was centrifuged at 3000 × g for 20 min at 4 °C. The pellet was re-suspended in a low salt buffer (20 mM Hepes, pH 7.8, 20 mM KCl, 1.5 mM MgCl_2_, 25 % glycerol, 200 mM EDTA, 500 mM DTT, and 200 mM PMSF) and centrifuged at 3000 × g for 10 min at 4°C (this step was repeated for 2 times). The pellet was suspended in a high salt buffer (20 mM Hepes, pH 7.8, 1 M KCl, 1.5 mM MgCl_2_, 25 % glycerol, 200 mM EDTA, 500 mM DTT, and 200 mM PMSF) in a volume of the estimated PNV (pellet nuclear volume). The nuclear lysate was incubated for 30 min at 4 °C with shaking and then centrifuged at 12 000 × g for 20 min at 4 °C. The supernatant was dialyzed in the low salt buffer for over 1 h. Finally, the protein content was measured by the Coomassie Brilliant Blue method. The EMSA was conducted as described by Hellman [50]. Probes that covered the *Monkey King* sequence were synthesized (Additional file 4).

### Vector construction, plant transformation, and GUS activity analysis

The *Monkey King* sequence was amplified from a *B. napus* BAC (JBnB75D10) carrying the *Monkey King* insertion in the *BnA10.FLC* promoter [31] using specific primers (Additional file 5) and cloned into the *Hin*dIII site of the binary vector pBI121 (Clontech, USA). The correct orientation was confirmed by DNA sequencing. The original pBI121 vector was used as a control. The two vectors were introduced into *Agrobacterium tumefaciens strain* LBA4404 and then transformed into *A. thaliana* using the floral dip method [51]. A segregation assay was used to identify lines harboring a single transgene copy, by culturing the T_2_ transgenic plants on half-strength MS medium containing 50 mg L^−1^ of kanamycin. T_3_ lines carrying a single transgene copy were assumed to be homozygous lines when they did not segregate for kanamycin resistance.

T_3_ homozygous lines were used to study the effect of the *Monkey King* element on gene expression. Ten-day-old seedlings grown on MS medium were harvested for RNA collection and GUS staining. Total RNA was extracted from about 25 seedlings using a Universal Plant Total RNA Extraction Kit (Cat.# RP3301, BioTeke, Beijing, China). RNA samples were treated with DNaseI and used for reverse transcription using M-MLV reverse transcriptase (Promega). SYBR-Green qRT-PCR was used to quantify the transcription level of the GUS gene in transgenic seedlings. The 25 μL reaction contained 12.5 μL SYBR Green PCR Master Mix (Toyobo), 600 nM primers, and 2 μL of 5 × diluted cDNA sample. The PCR profile was as follows: 95 °C for 1 min, followed by 40 cycles of 95 °C for 5 sec and 60 °C for 1 min. Fluorescence data were collected after the 60 °C step. Three replicate reactions were performed with each cDNA sample and individual primer pairs (Additional file 5). Chemical staining for GUS activity was performed as described by Jefferson et al. [52] with minor modifications. Seedlings were soaked in the GUS assay solution and incubated at 37 °C for 24 h. The GUS assay solution contained 1 g L^−1^ X-Gluc, 0.5 mM K_3_Fe(CN)_6_, 0.5 mM K_4_Fe(CN)_6_, 10 mM Na_2_EDTA, 0.1 % (v/v) Triton X-100, 20 % (v/v) methanol, and 100 mM sodium phosphate (pH 7.0). After staining, the samples were rinsed with ethanol and photographed.

### Availability of supporting data

Phylogenetic data is available in the TreeBASE as accession number S17406 (http://purl.org/phylo/treebase/phylows/study/TB2:S17406 ).

## Additional files

Additional file 1:
**Physical position and annotation of 504 complete sequences of** ***Monkey King*** **members on the** ***B. rapa*** **genome.**
Additional file 2:
**Physical position and annotation of 38 complete sequences of** ***Monkey King*** **members on the A. thaliana genome.**
Additional file 3:
**Characterization of the significant similarities obtained from BLASTN searches using** ***Monkey King*** **as the query against** ***Brassica*** **EST database from NCBI.**
Additional file 4:
**Sequences of probes used in EMSA.**
Additional file 5:
**Sequence information for primers used for** ***Monkey King*** **insertion polymorphisms analysis, DNA methylation analysis, vector construction and GUS gene expression analysis.**

## Abbreviations

MITE: Miniature inverted repeat transposable element
EST: Expressed sequence tag
TIR: Terminal inverted repeats
TSD: Target site duplications
FLC: FLOWERING LOCUS C
EMSA: Electrophoretic mobility shift assay
UTR: Untranslated region
qRT-PCR: Quantitative real-time reverse transcription PCR
